# Factors affecting UK anaesthetic trainees' wellbeing and stress: a scoping review

**DOI:** 10.1111/anae.16410

**Published:** 2024-09-10

**Authors:** Sophie Winter, Nicola Brennan, Thomas Gale

**Affiliations:** ^1^ Collaboration for the Advancement of Medical Education Research, Peninsula Medical School, Faculty of Health University of Plymouth Plymouth UK; ^2^ University Hospitals Plymouth NHS Trust Plymouth UK

**Keywords:** anaesthetist, stress, trainee, wellbeing

## Abstract

**Background:**

Poor wellbeing and stress in UK anaesthetic trainees impacts significantly on clinical performance, workforce retention and patient care. This study aimed to provide an overview of the evidence in this field and to explore the factors affecting wellbeing and stress in UK anaesthetic trainees.

**Methods:**

MEDLINE, Embase, PsycINFO, and ERIC were searched, in addition to organisational websites. Literature reporting factors affecting wellbeing and stress in UK anaesthetic trainees from 2009 to present were included.

**Results:**

Following exclusions, 45 studies were identified. Only five papers included qualitative analyses. Within these studies, 28 different phenomena related to wellbeing and stress were investigated. Thirty‐one different factors affecting anaesthetic trainees' wellbeing and stress were identified in this review. These have been summarised as individual; training; clinical role; progression; work patterns; resources; rest; support; and cultural factors. External factors were described as affecting wellbeing and stress more frequently than internal factors. The most frequently cited individual factors were fatigue and pre‐existing health status.

**Conclusions:**

The wide scope of phenomena of interest and measurement tools emphasises the challenge of defining and researching the concept of wellbeing. Despite these limitations, we have created a novel conceptual model of individual and external factors affecting UK anaesthetic trainees' wellbeing and stress. This supports an increased awareness and understanding of these factors, so that improvements can be made to practice and policy.

## Introduction

The recent NHS performance statistics are a sobering reflection of a healthcare “*deep in crisis*” [1] where growing clinical pressures, insufficient funding, understaffing and poor retention have led to concerns about its future [2]. A central issue is increasing burnout in healthcare workers, coupled with decreasing work satisfaction and motivation [3, 4]. In response, the mental wellbeing of staff has been identified as a key target in the NHS long‐term workforce plan. However, wellbeing is a nebulous concept to define and understand, which can make research and improvement challenging. Therefore, it is often used synonymously with other concepts such as health [5] or in the context of negative states, such as stress.

Anaesthetic trainees report high levels of stress, burnout and depression [6, 7, 8] which have been associated with a reduced ability to work effectively in the clinical environment and manage non‐clinical commitments. This has deleterious effects on performance, patient safety and trainees' own health [9, 10, 11, 12, 13]. Poor wellbeing has led some trainees to consider leaving anaesthetic practice or the NHS altogether, with only half of UK anaesthetic trainees planning on working in the NHS for their entire career, and a quarter planning to leave within 5 years [14]. Poor workforce retention is a significant concern in anaesthetics as the current shortfall of 1400 UK anaesthetists is projected to increase to 11,000 by 2040 if changes are not made [10]. As the future of the speciality relies on the next generation of anaesthetists, supporting trainee wellbeing is key to maintaining a safe and sustainable workforce. It is therefore imperative to understand the factors contributing to anaesthetic trainees' wellbeing and stress.

Wellbeing has a growing body of literature, yet there is no clear consensus as to how to define it [5]. The phrase appears ubiquitously within the World Health Organization definitions of both mental health and health, but this synonymous use has been criticised for making research and comparison challenging [15]. Equally, wellbeing is considered as a positive concept, but research often focuses on negative states, such as depression or stress, which are more easily identified and measured [16]. These assessments are based on a hedonic perception of wellbeing, described as the presence of pleasure and absence of pain. Alternative eudemonic approaches consider whether individuals are functioning well and achieving their potential, through assessments of confidence, performance and preparedness [16].

Healthcare workers are exposed regularly to potential stressors, and psychological stress occurs when these situations exceed an individual's resources [17]. Burnout may occur if stress continues without adequate recovery and is recognised through a triad of emotional exhaustion, depersonalisation and a reduced sense of personal accomplishment [18, 19]. In the wider healthcare community, workforce wellbeing concerns have led to the development of conceptual frameworks. These aim to support individuals and organisations to improve this issue.

The *Caring for Doctors, Caring for Patients* review described an ABC approach to doctors' core work needs based on autonomy, belonging and competence [20]. Brigham et al. developed a more exhaustive conceptual framework organised into individual and external factors [21]. However, before this review, there has been no framework developed specifically for anaesthetic trainees. This is needed as there are distinct differences between speciality roles and training pathways which affect trainee wellbeing and stress. This project aimed to address this gap by exploring the existing research and identifying factors through a scoping review of the literature. The conceptual model created from this work will help to inform educators, policy makers and individuals in efforts to improve trainee wellbeing and stress and to guide future research.

## Methods

A scoping review was chosen specifically to provide an overview of the evidence in this field, highlight knowledge gaps and reveal emerging concepts [22, 23]. This is particularly valuable for heterogenous data, as was anticipated from a preliminary literature review. This methodological choice was tested against the scoping review decision‐making flowchart by Pollock et al. [24] with a positive outcome. The Joanna Briggs Institute framework [22] was followed and review reported against the PRISMA‐SCr checklist [25].

The search strategy was created using a population concept context framework and peer reviewed by an experienced information specialist. We searched MEDLINE, Embase, PscyINFO and ERIC to identify clinical and educational literature (online Supporting Information Appendix S1). Grey literature searching was undertaken through ETHOS and we searched key stakeholder websites from the Royal College of Anaesthetists, Association of Anaesthetists and General Medical Council. All searches were performed on 4 March 2023, and backwards citation searching was performed on included studies.

We applied the following inclusion criteria: studies in the English language involving anaesthetic trainees; factors affecting wellbeing, stress and related concepts such as burnout, fatigue and mental health; and publication date 2009 to present (after the most recent changes to the European Working Time Directive). Due to differences in training structure and lifestyle between countries, a validated UK search filter was used [26, 27]. There were no limitations set on the study type to increase the breadth of data collected and all outcome measures were considered. Identified papers were exported into EndNote (Clarivate, London, UK) and automatic software was used to recognise duplicates, which were manually reviewed and removed. The remaining papers were screened using Rayyan QCRI software [28] against the inclusion criteria, and further manual deduplication occurred at this stage.

Papers were assessed systematically using a standardised selection form which was trialled before use (online Supporting Information Appendix S2). The title and abstracts of all papers were screened, proceeding to full text screening if inclusion was unclear. For papers where trainee inclusion was not clear, authors were contacted to confirm this. A total of 10% of identified studies were blindly reviewed with a high level of agreement, with any differences in inclusion discussed and agreed.

A data extraction form was developed (online Supporting Information Appendix S3), building on the framework of individual and external factors affecting clinician wellbeing by Brigham et al. [21]. We also added further categories including the type of data analysis performed and reclassified organisational and work‐related factors to external. Critical appraisal is not obligatory for scoping reviews unless specifically indicated to achieve the review objectives [22, 25]. As this project aimed to explore the scope of factors and research, as opposed to appraising the quality of the current literature, formal critical appraisal was not performed. A proposed strength of this approach is that a greater range of studies can be reviewed without the limitations of quality evaluation [29]. The data extraction for each included study was completed independently by SW with 10% being completed blindly by TG to check for comprehensive and consistent extraction.

The results were tabulated and organised further by frequency distribution of defined categories and NVivo 14 [30] was used to help identify key themes. A concern of synthesising primary studies is that findings may be de‐contextualised which can decrease their validity [31]; therefore, a textual narrative synthesis was used to combine the heterogenous qualitative and quantitative data into more homogenous groups, while still identifying study characteristics, highlighting diversity and revealing evidence gaps [32]. This has been used successfully in previous scoping reviews [33, 34].

## Results

**Figure 1:**
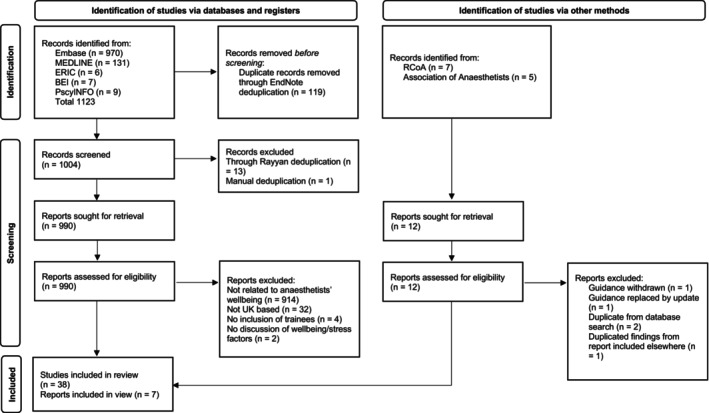
Study flow chart. BEI, British Education Index; GMC, General Medical Council; RCoA, Royal College of Anaesthetists.

The literature search identified 1123 papers, of which 45 were included in the review (Fig. 1) with full results detailed in online Supporting Information Appendix S4.

A summary of study characteristics is shown in Table 1. Cross‐sectional observational studies were the most common project design (26/45) and qualitative or mixed methods surveys were the most frequent data collection tool (31/45). There were a small number of purely qualitative studies (4/45) which were performed using interviews and thematic analysis. Qualitative results from mixed‐methods studies were gained from questionnaires with free‐text open questions. Eight studies were expert consensus or editorial pieces which did not report any primary data.

**Table 1:** Summary of characteristics of included studies.

Characteristic	Category	Number of studies
**Study location**	Local	18
	National	14
	Regional	3
	Multi‐region	2
**Participant numbers**	≤ 20	7
	21–100	10
	101–500	6
	501–1000	2
	1001–5000	7
	> 5000	1
**Study design**	Cross‐sectional observational	26
	Editorial	5
	Prospective longitudinal observational	5
	Qualitative phenomenological	4
	Expert consensus	3
	Pre‐post interventional	1
	Case report	1
**Data collection**	Survey	17
	Mixed‐methods survey	14
	Interviews	4
	Physiological observations	1
	Focus groups	1
	Other	9
**Data type**	Quantitative	17
	Qualitative and quantitative	15
	Opinion	6
	Qualitative	4
	Expert consensus	3
**Data analysis**	Descriptive	29
	Thematic analysis	5
	Statistical	4
	Exploratory sentiment analysis	1
	Other	9

Twenty‐eight concepts were identified in the literature with the most frequent being wellbeing; stress; burnout; morale; and fatigue. Some studies took a eudemonic approach to the issue by considering whether participants were functioning well and achieving their potential, through confidence, performance and preparedness. Eleven different pre‐existing tools were used to measure these concepts, of which only the GHQ‐12 [35] and IES‐R [36] were used more than once across a series of prospective longitudinal studies. Participant self‐reported measures were used in 26/45 studies.

Thirty‐one factors affecting anaesthetic trainee wellbeing and stress were identified, which were classified into nine main categories: individual; training; clinical role; progression; work patterns; resources; rest; support; and culture. Most identified factors were external and were reported as adversely impacting wellbeing or contributing to negative states such as stress.

**Figure 2:**
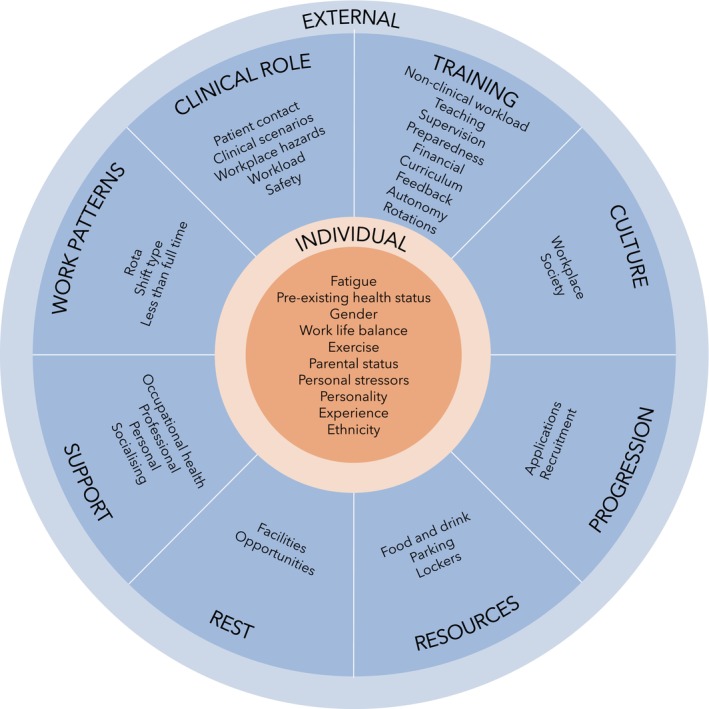
Conceptual framework of factors affecting UK anaesthetic trainees' wellbeing and stress.

Due to the number and variety of results, a visual framework was created to summarise the key findings from this review. We considered adapting the model by Brigham et al. [21]; however, this places patient wellbeing and the clinician‐patient relationship at its core and does not reinforce the interaction between external and internal factors. An alternative model used by Warren et al. [37] was Maslow's hierarchy of needs [38]. An attempt was made to replicate this approach but not all identified factors were incorporated, while others appeared more than once. Equally the hierarchical order and lack of social factors in this model has been critiqued [39]. Therefore, a novel framework (Fig. 2) was created. This places individual factors at the centre of trainee wellbeing, surrounded by external factors to highlight their interaction. The analysis of the primary studies supported a bi‐directional approach to wellbeing and stress, whereby the presence or absence of a factor either supported wellbeing or increased negative states such as stress. Therefore, identified factors have been summarised together in the framework, accompanied by a detailed narrative summary.

Fatigue was the most frequently described individual factor that contributed to stress, risk of suicide and negatively impacted trainee wellbeing [12, 13, 40, 41, 42, 43, 44, 45, 46, 47]. Fatigue is affected by personal behaviours such as sleep hygiene but is also significantly impacted by external factors such as long hours, on‐calls, night shift work and a lack of rest facilities. Pre‐existing mental or physical ill‐health, low exercise levels, a perfectionist personality type and not being a parent were other factors associated with poor wellbeing, stress and a risk of suicide [6, 13]. Clinical inexperience was related to increased stress, particularly noted in obstetric anaesthesia [48] and during the COVID‐19 pandemic [49]. However, experienced registrars have been found to have lower wellbeing scores than core trainees [50]. Pregnancy and ageing were noted to impact workplace ergonomics, safety and health [51], while ethnicity has been identified as a risk factor for COVID‐19‐related trauma [52].

The most common mode of suicide in the anaesthetist cohort is anaesthetic medications and having access to controlled drugs has been identified as a risk factor for substance use disorder and suicide [13, 53]. Individual risk factors include personal stressors such as criminal or regulatory body investigations or relationship issues. The suicide risk related to male or female sex in anaesthetists reflects statistics from the general population, with a ratio of 5.6:1 male vs. female. Male anaesthetic trainees appear to have a higher risk of burnout while females are more likely to perceive stress [6]. Finally, a poor work–life balance was reported to both lead to, and be a result of, decreased morale and welfare [7, 14, 54].

Training aspects were the most referenced external factor impacting wellbeing and stress, followed closely by those related to the anaesthetic clinical role. Different subfactors ranged from the local learning environment to how training is organised. Frequent rotations were raised as a negative factor, associated with long commutes, social isolation and a perceived lack of autonomy in training [7, 37, 54, 55]. Within the learning environment, trainees reported the benefits of autonomous practice when performed with appropriate safe supervision, but the tension between service provision and training was noted [7]. The one‐to‐one learning environment of anaesthesia has been praised but a lack of senior support was a reported issue for night shifts and obstetric anaesthesia specifically [46, 48, 56]. Intensive care unit (ICU) and on‐call shifts appear to have low levels of trainee preparedness [57] and this has also been reported as an issue for trainees returning to clinical work after a period of absence [58]. Induction programmes can be an effective strategy to increase preparedness and reduce anxiety, in addition to structured teaching programmes [37, 57, 59].

The burden of the non‐clinical workload, number of assessments and a perceived tick box approach were identified [7, 50, 54, 55, 60, 61]. The 2021 Royal College of Anaesthetists' (RCoA) curriculum update responded to these concerns by moving to more holistic assessments of competencies; however, the transfer between curricula has itself contributed to increased stress [54, 62]. The anaesthetic speciality examination is a source of anxiety for many trainees and has been described as more difficult compared with other specialties and not relevant to training [7, 13, 61]. Additionally, the time commitment to revision has been associated with burnout and depression risk, while trainees feel that their examination performance is impacted by fatigue [6, 12]. Furthermore, the financial cost of training, including examination fees, has been noted to negatively impact trainee wellbeing [7]. This is in the wider context of perceived pay cuts [11, 55] and a lack of remuneration for additional clinical work during the pandemic [63].

Issues with training progression and the recruitment application process were raised [61, 64]. Changes to self‐scoring portfolio assessments during the pandemic were seen as unfair by trainees [54] and, although interviews have been reintroduced virtually, recruitment has not yet returned to pre‐pandemic processes. In addition, exam cancellations and disrupted training opportunities led to a backlog of trainees unable to progress [61] and this has been exacerbated by the 2021RCoA curriculum changes. Worries over the lack of registrar jobs has left 75% of trainees not confident that they will secure a post [54]. Equally, trainees reported the additional stress of organising a top‐up year if they were unable to secure an speciality training post in the final recruitment round.

The clinical role of anaesthetic trainees can have positive impacts on wellbeing through enabling good outcomes via patient contact and learning skills [14, 55]. However, a high workload intensity was reported across general anaesthesia, intensive care and obstetric anaesthesia [43, 48, 59]. Stressors specific to obstetric anaesthesia include high acuity clinical scenarios, while more universal clinical stressors include system pressures, a lack of intensive care beds, critical incidents, complaints and feeling unsafe [7, 41, 55, 65, 66]. The impact of workplace hazards [51] has been raised, notably with respect to the infection risk and lack of personal protective equipment during the COVID‐19 pandemic [37, 49, 52, 63, 67, 68]. The pandemic had a wider negative impact on the workforce, with 30% of trainees less likely to stay in the NHS due to feeling under‐appreciated, under‐supported, over‐worked and experiencing trauma at work [14].

Within local departments, rota issues were reported commonly. These include late provision; last minute changes; inadequate staffing; difficulties taking annual or study leave; and a lack of rota flexibility [6, 7, 14, 41, 49, 55]. On‐call and night work, in addition to the number, irregularity, intensity and length of shifts, were described as negatively impacting wellbeing, stress and fatigue [11, 37, 46, 47, 49, 50, 56, 60, 63]. This has led to appeals for less on‐call work, shorter shifts and adequate rest times, while several trainees are considering less‐than‐full‐time training to improve work–life balance [7, 14, 40].

There were reports of inadequate rest facilities, with limited time for breaks within and between shifts. This is in addition to negative attitudes towards trainees resting on night shifts [7, 41, 43, 44, 69]. Wider cultural issues raised include the societal shift in the view of doctors and the impact of the junior doctor disputes [7, 11]. Within healthcare organisations, a general negative culture was described, in addition to specific concerns regarding politics, poorly functioning teams, bullying and harassment [7, 50, 53, 64]. Trainees reported feeling under‐appreciated and under‐valued, with a perceived imbalance of effort with reward [6, 7, 12, 14, 44, 54, 70]. A lack of support from departments, colleagues and Trusts was reported in some cases [14, 53, 64]; however, there were also positive reports of anaesthetic departments and seniors being supportive, welcoming and friendly [7, 41]. Proactive leadership from senior clinicians and high levels of departmental engagement have been reported to reduce burnout and improve wellbeing [37, 51, 71], in addition to support from friends, family and colleagues [7, 13, 37, 41, 55, 64, 72]. Limited opportunities to meet and share experiences with peers have been raised [73] but this can be encouraged by professional social initiatives such as ‘coffee and a gas’ and debriefs [64]. Other initiatives such as mindfulness programmes were described but it has been argued these are not as effective as targeting core issues [54].

The final external factor was the provision of resources to meet trainees needs. A lack of lockers was identified as a negative factor, whereas free parking and food during the pandemic contributed positively to workforce wellbeing [7, 37, 40]. Despite guidance that refreshments should be available at all times [42], there is a reported lack of availability out of hours [7].

## Discussion

A structured scoping review of the literature was performed which has improved rigour compared with frameworks created by collaborative working groups. This has enabled identification of pressures that UK anaesthetic trainees face from non‐clinical workloads, clinical roles such as ICU and progression issues, combined with the financial and rotational burden of training. National organisations such as the RCoA, Association of Anaesthetists, Health Education England and General Medical Council should therefore consider the significant influence they have on trainee wellbeing through guidance and policies. This is in the context of national industrial action for pay restoration [74] and the 2023 RCoA Extraordinary General Meeting where resolutions were proposed regarding anaesthetic recruitment and rotations [75]. Additional proposals were raised regarding the impact of the expansion and supervision of anaesthesia associates, but these factors have not been detailed in the literature from this review.

Regionally, practical changes to support wellbeing include the provision of rest facilities, resources such as lockers and professional support services. Informal support systems can be encouraged through departmental social initiatives and a positive workplace culture. Rotas should be designed to support adequate rest and leave opportunities and departments should ensure that trainees are clinically supported through appropriate supervision arrangements, induction processes and management of workplace hazards. Individual trainee factors have been highlighted which can support identification of individuals at risk of poor wellbeing and burnout, such as those with pre‐existing health issues, personal stressors and perfectionist personality types. Trainees are also encouraged to reflect on their personal experiences and consider actions to improve wellbeing such as exercise and good sleep hygiene.

A scoping review methodology was chosen to provide an overview of evidence and reveal emerging concepts, but the breadth of this approach can be at the expense of depth. In this review, the search strategy was peer‐reviewed by an information specialist to encourage rigour, and steps have been taken throughout the review to reduce bias. The inclusion of editorials and cross‐sectional quality improvement projects is less rigorous than for a systematic review but supports our aim of exploring the current breadth of research.

Within the included primary research studies there were a significant number of different measurement tools used, despite recommendations to use previously validated instruments where possible [76]. A frequent issue was limited transparency and reporting of rationale, instrument development and analysis. Most of the literature reported solitary, small local research projects, using quantitative or mixed‐methods cross‐sectional surveys. For many identified factors, there was no qualitative exploration as to how they affect trainee experiences; an increased in‐depth understanding would be a valuable future research focus.

Literature was limited to the UK, but international studies have identified factors which may have local relevance. These include clinical scenarios such as patients' pre‐operative physical status and operating theatre distractions, in addition to wider issues regarding gender inequality and harassment [77, 78, 79, 80]. Anaesthetists outside of training were excluded due to different commitments and potential stressors, but specific concerns have been raised regarding consultants and Staff and Associate Specialists [50, 81]. Further research and improvements for these cohorts are an important consideration.

Several factors affecting UK anaesthetic trainee wellbeing and stress have been identified and summarised in a novel conceptual model. This illustrates individual factors, related to trainee demographics and lifestyle, and external factors within the clinical, training and cultural environment. External factors were the influence described most frequently on wellbeing and stress, particularly with respect to training, the clinical role and work patterns, while individual influences, such as fatigue and pre‐existing health status, appeared less. There is a close association between factors such as work patterns and fatigue but the tension between competing demands like service provision and training has also been discussed. The identified factors have been reflected on to suggest areas for improvement in practice and policy at an individual, local and national level.

This evidence synthesis supports an awareness of the existing research in this field and recommends consistency and transparency in future research methodology. A qualitative research gap has been identified and ongoing work is needed to further explore these factors and to understand how supportive interventions work and in what context.

## Supporting information


**Appendix S1.** Scoping review search strategies.
**Appendix S2.** Abstract selection form.
**Appendix S3.** Data extraction form.
**Appendix S4.** Results.
