# Supplementary material for: Factors affecting UK anaesthetic trainees' wellbeing and stress: a scoping review
Source: Anaesthesia. 2024 Sep 10;80(2):151–60. doi: 10.1111/anae.16410 (PMC11726272; doi:10.1111/anae.16410)
Supplement: Supplementary file 1 — Appendix S1. Scoping review search strategies. Appendix S2. Abstract selection form. Appendix S3. Data extraction form. Appendix S4. Results. [file ANAE-80-151-s001.docx]

**Appendix S1:** **Scoping review search strategies**

Ovid MEDLINE(R) and Epub Ahead of Print, In-Process, In-Data-Review & Other Non-Indexed Citations, Daily and Versions (from 2010)

1. Exp mental health/
2. Exp mental disorders/
3. Esp stress, psychological/
4. Exp occupational stress/
5. Exp burnout, psychological/
6. Exp burnout, professional/
7. Exp anxiety/ or exp anxiety disorders/
8. Exp mood disorders/
9. Exp depression/ or exp depressive disorder/
10. Esp Substance-Related Disorders/
11. (suicide or stress* or distress or burnout or burn-out or anxiety or mood or depress*).ab,kw,ti.
12. ((mental or psychological) adj3 (health or ill*)).ab,kw,ti.
13. (wellness or well-ness or wellbeing or well-being).ab,kw,ti.
14. Addict*.ab,kw,ti.
15. ((substance or drug or alcohol) adj3 (misus* or use* or abuse*)).ab,kw,ti.
16. Or/1-15
17. Exp anesthetists/
18. Anaesthetist*.ab,kw,ti.
19. ((anaesth*) adj3 (doctor* or train*)).ab,kw,ti
20. Or/17-19
21. 16 and 20
22. Exp United Kingdom/
23. (national health service* or nhs*).ab,kw,ti.
24. (English not ((published or publication* or translat* or written or language* or speak* or literature or citation*) adj5 english)).ti,ab.
25. (gb or "g.b." or britain* or (british* not "british columbia") or uk or "u.k." or united kingdom* or (england* not "new england") or northern ireland* or northern irish* or scotland* or scottish* or (wales or "south wales") not "new south wales") or welsh*).ti,ab,kw,in.
26. (bath or "bath's" or (birmingham not alabama*) or (*birmingham's" not alabama*) or bradford or "bradford's" or brighton or "brighton's" or bristol or "bristol's" or carlisle* or "carlisle's" or (cambridge not (massachusetts* or boston* or harvard*)) or ("cambridge's" not (massachusetts* or boston* or harvard*)) or (canterbury not zealand*) or ("canterbury's" not zealand*) or chelmsford or "chelmsford's" or chester or "chester's" or chichester or "chichester's" or coventry or "coventry's" or derby or "derby's" or (durham not (carolina* or nc)) or ("durham's" not (carolina* or nc)) or ely or "ely's" or exeter or "exeter's" or gloucester or "gloucester's" or hereford or "hereford's" or hull or "hull's" or lancaster or "lancaster's" or leeds* or leicester or "leicester's" or (lincoln not nebraska*) or ("lincoln's" not nebraska*) or (liverpool not (new south wales* or nsw)) or ("liverpool's" not (new south wales* or nsw)) or (london not (ontario* or ont or toronto*)) or ("london's" not (ontario* or ont or 1706641 Advanced toronto*)) or manchester or "manchester's" or (newcastle not (new south wales* or nsw)) or ("newcastle's" not (new south wales* or nsw)) or norwich or "norwich's" or nottingham or "nottingham's" or oxford or "oxford's" or peterborough or "peterborough's" or plymouth or "plymouth's" or portsmouth or "portsmouth's" or preston or "preston's" or ripon or "ripon's" or salford or "salford's" or salisbury or "salisburys" or sheffield or "sheffield's" or southampton or "southampton's" or st albans or stoke or "stoke's" or sunderland or "sunderland's" or truro or "truro's" or wakefield or "wakefield's" or wells or westminster or "westminster's" or winchester or "winchester's" or wolverhampton or "wolverhampton's" or (worcester not (massachusetts* or boston* or harvard*)) or ("worcester's" not (massachusetts* or boston* or harvard*)) or (york not ("new york*" or ny or ontario* or ont or toronto*)) or ("york's" not ("new york*" or ny or ontario* or ont or toronto*))))).ti,ab,in.
27. (bangor or "bangor's" or cardiff or "cardiff's" or newport or "newport's" or st asaph or "st asaph's" or st davids or swansea or "swansea's").ti,ab, in.
28. (armagh or "armagh's" or belfast or "belfast's" or lisburn or "lisburn's" or londonderry or "londonderry's" or derry or "derry's" or newry or "newry's").ti,ab,in.
29. or/22-28
30. (exp africa/ or exp americas/ or exp antarctic regions/ or exp arctic regions/ or exp asia/ or exp australia/ or exp oceana/) not (exp United Kingdom/ or europe/)
31. 29 not 30
32. 31 and 27
33. 32 and 2010:2023.(sa_year).

PsycInfo: https://www.proquest.com/psycinfo/results/A4A680DE8BC344C4PQ/1?accountid=14711

(MAINSUBJECT.EXACT.EXPLODE("Substance Related and Addictive Disorders") OR MAINSUBJECT.EXACT.EXPLODE("Well Being") OR MAINSUBJECT.EXACT.EXPLODE("Burnout") OR MAINSUBJECT.EXACT.EXPLODE("Self-Destructive Behavior") OR MAINSUBJECT.EXACT.EXPLODE("Stress") OR MAINSUBJECT.EXACT.EXPLODE("Occupational Stress") OR MAINSUBJECT.EXACT.EXPLODE("Mental Health") OR MAINSUBJECT.EXACT.EXPLODE("Mental Disorders") OR ti(wellbeing OR well-being OR burnout OR burn-out OR stress OR anxiety OR mental OR depress*) OR ab(wellbeing OR well-being OR burnout OR burn-out OR stress OR anxiety OR mental OR depress*) ) AND (MAINSUBJECT.EXACT("Anesthesiology") OR Anaesthetist* OR (Anaesth* PRE/3 (train* OR doc*))) AND (af("United Kingdom" OR UK OR "U.K." Britain OR British OR England OR Scotland OR Scottish OR Ireland OR Irish OR Wales OR Welsh OR "National Health Service" OR NHS) OR ti("United Kingdom" OR UK OR "U.K." Britain OR British OR England OR Scotland OR Scottish OR Ireland OR Irish OR Wales OR Welsh OR "National Health Service" OR NHS) OR ab("United Kingdom" OR UK OR "U.K." Britain OR British OR England OR Scotland OR Scottish OR Ireland OR Irish OR Wales OR Welsh OR "National Health Service" OR NHS) OR lo.Exact("Great Britain" OR "Ireland" OR "Wales" OR "England" OR "United Kingdom" OR "Scotland" OR "Northern Ireland")) AND pd(20100101-20240101)

Embase 1974 to 2023 March 01

1. mental disease/
2. wellbeing/
3. mental stress/
4. job stress/
5. professional burnout/
6. burnout/
7. mood disorder/
8. depression/
9. addiction/
10. anxiety disorder/
11. (wellness or well-ness or well-being or wellbeing).ab,kw,ti.
12. (mental or psychological) adj3 (health or ill).ab, kw,ti.
13. (suicide or stress* or distress or burnout or burn-out or anxiety or mood or depress" or addict*).ab, kw, ti.
14. (substance or drug or alcohol) adj3 (misus" or use* or abuse*)).ab,kw,ti.
15. or/1-14
16. exp anesthetists/
17. anaesthetist*.ab,kw,ti.
18. (anaest" adj3 (doctor" or train*)).ab,kw,ti.
19. ог/16-18
20. 15 and 19
21. United Kingdom/
22. Great Britain/
23. Ireland/
24. Northern Ireland/
25. (national health service* or NHS*).ab,ad,in,ti.
26. (gb or "g.b." or britain" or (british* not "british columbia").ab, ad, in,ti.
27. (UK or "U.K." or United Kingdom*).ab,ad,in,ti.
28. (England" not "new England").ab,ad, in, ti.
29. (Ireland or Irish or Scotland or Scottish or (Wales or "South Wales") not "new South Wales") or Welsh). ab,ad,in,ti.
30. (bath or "bath's" or (birmingham not alabama*) or ("birmingham's" not alabama*) or bradford or "bradford's* or brighton or "brighton's* or bristoi or 'bristol's" or carlisie* or "carlisie's' or (cambridge not (massachusetts* or boston* or harvard*)) or ("cambridge's" not (massachusetts* or boston* or harvard*)) or (canterbury not zealand*) or ("canterbury's" not zealand") or chelmsford or "chelmsford's" or chester or "chester's" or chichester or "chichester's" or coventry or "coventry's"| or derby or "derby's" or (durham not (carolina* or nc)) or ("durham's" not (carolna* or nc)) or ely or "eiy's" or exeter or "exeter's" or gioucester or "gloucester's" or hereford or "hereford's" or hull or "hull's" or lancaster or "lancaster's" or leeds* or leicester or "leicester's" or (lincoln not nebraska*) or ("lincoin's' not nebraska* or liverpool not (new south wales* or nsw)) or ("liverpool's" not (new south wales* or nsw)) or (london not (ontario* or ont or toronto*)) or ("london's" not (ontario* or ont or toronto*)) or manchester or "manchester's" or (newcastie not (new south wales* or nsw]) or ('newcastle's" not (new south wales* or nsw)) or norwich or "norwich"s" or nottingham or "nottingham's" or oxford or "oxford's" or peterborough or "peterborough's" or plymouth or "plymouth's" or portsmouth or "portsmouth's" or preston or "preston's" or ripon or "ripon's" or salford or "salford's" or salisbury or "salisbury's* or sheffield or "sheffield's" or southampton or "southampton's" or st albans or stoke or "stoke's" or sunderland or "sunderland's" or truro or "truro's" or wakefield or 'wakefield's" or wells or westminster or "westminster's" or winchester or "winchester's' or wolverhampton or "wolverhampton's" or (wordester not (massachusetts* or boston* or harvard*)) or ("worcester's" not (massachusetts" or boston* or harvard*)) or (york not ("new york" or ny or ontario* or ont or toronto*)) or ("york's" not ("new york"" or ny or ontario* or ont or toronto*).,ab,ad, in, ti.
31. (bangor or "bangor's" or cardiff or "cardiff's" or newport or "newport's" or st asaph or "st asaph's" or st davids or swansea or "swansea's").ab,ad,in,ti.
32. (aberdeen or "aberdeen's" or dundee or "dundee's" or edinburgh or "edinburgh's" or glasgow or 'glasgow's" or inverness or (perth not australia*) or ("perth's" not australia*) or stirling or "stirling's*).ab,ad, in,ti.
33. (armagh or "armagh's" or belfast or "belfast's" or lisburn or "lisburn's" or londonderry or "londonderry's" or derry or "derry's" or newry or "newry's").ab,ad,in,ti.
34. or/21-33
35. (exp "arctic and antarctic" / or exp oceanic regions/ or exp western hemisphere/ or exp africa/ or exp asia/ or exp australia and new zealand*/) not (united kingdom/ or europe/)
36. 34 not 35
37. 20 and 36
38. 37 and 2010:2024.(sa_year).

ERIC and British Education Index

S1. TI anaesth* OR AB anaesth OR SU anaesth

S2. TI mental health OR AB mental health OR SU mental health

S3. TI ( burnout or burn-out or burn out or stress or occupational stress or compassionate fatigue ) OR AB ( burnout or burn-out or burn out or stress or occupational stress or compassionate fatigue ) OR SU ( burnout or burn-out or burn out or stress or occupational stress or compassionate fatigue )

S4. TI ( wellness or wellbeing or well-being or well being ) OR AB ( wellness or wellbeing or well-being or well being ) OR SU ( wellness or wellbeing or well-being or well being )

S5. TI suicide OR AB suicide OR SU suicide

S6. TI ( substance abuse or substance use or drug abuse or drug addiction or drug use ) OR AB ( substance abuse or substance use or drug abuse or drug addiction or drug use ) OR SU ( substance abuse or substance use or drug abuse or drug addiction or drug use )

S7. S2 OR S3 OR S4 OR S5 OR S6

S8. S1 and S7

ETHOS British library thesis

No results

Organisation search

| Search | Papers | Decision |
| --- | --- | --- |
| Site:rcoa.ac.uk “wellbeing” “burnout” “mental health” “stress” | RCOA Welfare and Morale report | Included |
|  | RCOA views from the frontline of anaesthesia during covid 19 April 2020 survey results | Included |
|  | RCOA views from the frontline of anaesthesia during covid 19 May 2020 survey results | Included |
|  | RCOA views from the frontline of anaesthesia during covid 19 July 2020 survey results | Included |
|  | Respected, valued, retained | Included |
|  | AAGBI Anaesthesia: national survey of the effects of fatigue on trainees in anaesthesia in the UK 2019 | Duplicate of search results |
|  | State of the nation report | Excluded: report of findings from respected valued retained |
|  |  |  |
| Site:anaesthetists.org  “wellbeing” “burnout” “mental health” “stress” | Substance use disorder in the anaesthetist | Excluded: identified in database search |
|  | Suicide amongst anaesthetists | Included |
|  | Fatigue and anaesthetists | Included |
|  | Drug and alcohol abuse amongst anaesthetists. Guidance on identification and management | Excluded: replaced by substance use disorder guidance. |
|  | Occupational health in the anaesthetist | Excluded: withdrawn guidance |
| Site:gmc-uk.org “anaesthetist” “wellbeing” “burnout” “mental health” “stress” | - |  |

**Appendix S2: Abstract selection form**

**Abstract selection form:** Factors affecting UK trainee Anaesthetists’ wellbeing and stress: a scoping review.

Each abstract to be independently appraised by one reviewer. 10% to be cross checked.

**Article details**

| **Title** |  |
| --- | --- |
| Year |  |
| Authors |  |
| Journal |  |

**Reviewer details**

| **Reviewer 1** | **Reviewer 2** |
| --- | --- |
|  |  |

**From reviewing the abstract, does it meet the criteria for this scoping review?**

| **Criterion** | | **Yes** | **No** | **Unclear** |
| --- | --- | --- | --- | --- |
| Population: | Are they trainee anaesthetists? |  |  |  |
|  | Are they in the UK? |  |  |  |
| Phenomenon of interest: | Is it regarding wellbeing/mental health/stress or related phenomena? |  |  |  |
|  | Is there discussion of factors related to this? |  |  |  |
| Study: | Does it include data (of any type: qualitative, quantitative, opinion)? |  |  |  |

IF Yes to all questions = INCLUDE

IF No to any questions = REJECT

IF Unclear to any questions = Obtain full text article and complete section below

**On reviewing the full text, does it meet the criteria for this scoping review?**

| **Criterion** | | **Yes** | **No** | **Unclear** |
| --- | --- | --- | --- | --- |
| Population: | Are they trainee anaesthetists? |  |  |  |
|  | Are they in the UK? |  |  |  |
| Phenomenon of interest: | Is it regarding wellbeing/mental health/stress? |  |  |  |
|  | Is there discussion of factors related to this? |  |  |  |
| Study: | Does it include data (of any type: qualitative, quantitative, opinion)? |  |  |  |

IF Yes to all questions = INCLUDE

IF No to any questions = REJECT

IF Unclear to any questions = final review of full text by third independent reviewer

IF Unclear to any questions from third reviewer = REJECT

**Final Decision**

| **Accept** | **Reject** |
| --- | --- |
|  |  |

**Signatures**

| **Reviewer 1** | **Reviewer 2** |
| --- | --- |
|  |  |

**Comments (optional)**

| **Reviewer 1** | **Reviewer 2** |
| --- | --- |
|  |  |

**Appendix S3: Data extraction form**

**Data collection form v2:** Factors affecting UK trainee Anaesthetists’ wellbeing and stress: a scoping review.

**Article details**

| **Title** |  |
| --- | --- |
| **Year** |  |
| **Authors** |  |
| **Journal** |  |

**Methods**

| **Type of study**  *Survey, case study, cross sectional, cohort, RCT, ethnography* |  |
| --- | --- |
| **Length of study**  *Single time point, prospective* |  |
| **Data collection**  *Questionnaires, interviews, focus groups* |  |
| **Type of data**  *Qualitative, quantitative, mixed methods* |  |
| **Data analysis used**  *Thematic analysis, descriptive* |  |

**Population**

| **Training level of anaesthetist**  *Core trainee, registrar, Trust grade* |  |
| --- | --- |
| **Number of participants**  *N= (N= any subgroups)* |  |

**Phenomenon of interest**

| **Stressors** | |
| --- | --- |
| **Concept identified**  *Burnout, suicide, substance misuse etc.* |  |
| **Instrument/tool used to measure concept**  *Validated instrument, self-reported etc.* |  |
| **Impact of phenomenon of interest**  *Time out of training, sickness leave, leaving training* |  |
| **Negative factors** | |
| **Individual** |  |
| **Organisational** |  |
| **Protective factors** | |
| **Individual** |  |
| **Organisational** |  |

**Appendix S4:** **Results**

| **Article** | | **Methods** | | | | | **Population** | | | **Phenomenon of interest** | | **Negative factors** | | **Protective factors** | |
| --- | --- | --- | --- | --- | --- | --- | --- | --- | --- | --- | --- | --- | --- | --- | --- |
| **Details** | **Journal** | **Study type** | **Length** | **Collection** | **Data type** | **Analysis** | **Level** | **No.** | **Type** | **Measure** | **Impact** | **Internal** | **External** | **Internal** | **External** |
| Harrison, J. The ailing anaesthetist. 2014. | Anaesthesia | Editorial | N/A | N/A | Opinion | - | All levels | - | Wellbeing Health | - | - | Pregnancy, fatigue, musculoskeletal issues, mental health. | Workplace hazards: inoculation injury, BBV, respiratory viruses, ionising radiation | High levels of engagement (motivation, involvement, advocacy) | Occupational health engagement, supportive culture |
| Tribe, I. The educational value of nightshifts: a perspective from anaesthetic trainees. 2023. | Anaesthesia: Conference abstract | Qualitative phenomenological  Local | Not described | Semi-structured interviews  Creation not described | Qualitative | Content thematic analysis | ST2-ST6 | 6  No denominator | Wellbeing | Self-reported | - | Fatigue | Nightshifts: lack of senior presence | - | - |
| Mohamad, A. and Valap, S. Impact of COVID-19 pandemic on mental health among anaesthetic trainees at Kettering General Hospital. 2021. | Anaesthesia: Conference abstract | Cross sectional observational  Local | ‘Before pandemic and after March 2020 lockdown’ | Survey  Creation not described | Quantitative | Descriptive | FY1 – SPR | 16  No denominator | Mental health | Self-reported | 63% reduction in motivation  38% looking forward to work (87.5% pre pandemic)  42.5% increase in sleeping difficulties | - | Covid-19 pandemic | - | - |
| Currie, C. et al.. Improving the return to clinical practice: A survey of current experience. 2018 | Anaesthesia: Conference abstract | Cross sectional observational  Local | Not described | Survey  Creation not described | Quantitative | Descriptive | Trainees (specific levels not discussed) | 60  No denominator | Confidence | Self-reported | - | - | Prolonged work absence (maternity leave, sick leave, out of programme, academic), lack of prereturn planning and support | - | - |
| Holmes, A. and Greatorex, B.. Improving wellbeing and reducing anxiety among core trainees in ICU via feedback led intervention. 2020. | Intensive Care Medicine Experimental: Conference abstract | Prospective longitudinal observational pre and post ITU bootcamp  Local | Not described | Survey  Creation not described | Quantitative | Descriptive | Core trainees | 7  No denominator | Anxiety  Preparedness | Self-reported | 100% of trainees reported reduction in anxiety and increase in preparedness with ITU bootcamp | - | Not feeling prepared for ITU and on calls, lack of standardised introduction | - | Induction |
| Hanna, A. et al. Intensive care medical staff wellbeing during the COVID-19 pandemic. 2022. | Journal of the Intensive Care Society: Conference abstract | Prospective longitudinal observational survey  Local | Once in December, again in April.  Time period otherwise not described. | Survey  Creation not described | Quantitative | Descriptive | Registrars, core trainees, foundation doctors, clinical fellows and advanced critical care practitioners. | 18 to first survey  15 to second survey  No denominator | Burnout | BMA burnout questionnaire | 56% reported high or very high levels of burnout in December compared to 47% in April | - | High workload, lack of break spaces | - | Teaching |
| Devlin, M. and Cassin, R. Joy ought to rule the day at work. 2019. | Anaesthesia: Conference abstract | Cross sectional observational  Local | Not described | Mixed methods survey  Creation not described | Quantitative and qualitative | Descriptive | Core trainees | 9/10 | Performance | Self-reported | - | - | Few opportunities to meet and share experiences with peers | - | Opportunities to socialise with peers |
| Roberts, T. et al. Psychological distress and trauma during the COVID-19 pandemic: survey of doctors practising anaesthesia, intensive care medicine, and emergency medicine in the United Kingdom and Republic of Ireland. 2021. | British journal of anaesthesia | Prospective longitudinal observational  National | 2 months | Survey  Iteratively adapted by research team, informed by literature review | Quantitative | Descriptive and statistical | Included junior and middle grade trainees | 1719 (701 anaesthetists, 298 junior/ middle grade)  No denominator. | Psychological distress  Trauma response | GHQ-12  IES-R | Psychological distress (GHQ-12 score >3) = 51.8% anaesthetics, 53.2% across all cohorts  Psychological trauma (IES-R >24) = 27.7% anaesthetics, 28.4% across all cohorts | - | Covid-19 | - | - |
| Roberts, T. et al. Psychological distress and trauma in doctors providing frontline care during the COVID-19 pandemic in the United Kingdom and Ireland: A prospective longitudinal survey cohort study. 2021. | BMJ Open | Prospective longitudinal observational  National | Acceleration: 1 week, peak: 2 weeks, deceleration: 2 weeks | Survey  Iteratively adapted by research team, informed by literature review | Quantitative | Descriptive and statistical | Included junior and middle grade trainees | 3079 (1114 anaesthetists, 506 junior/middle grade)  No denominator. | Psychological distress  Trauma response | GHQ-12  IES-R | Psychological distress (GHQ-12 score >3) = 32.6% anaesthetics, 31.5 % across all cohorts  Psychological trauma (IES-R >24) = 16.5% anaesthetics, 17.7% across all cohorts | Ethnicity as risk factor for covid related trauma | Covid-19, concerns about infection risk |  |  |
| Roberts, T. et al. Psychological distress during the acceleration phase of the COVID-19 pandemic: A survey of doctors practising in emergency medicine, anaesthesia and intensive care medicine in the UK and Ireland. 2021. | Emergency Medicine Journal | Prospective longitudinal observational  National | 8 days | Survey  Iteratively adapted by research team, informed by literature review | Quantitative | Descriptive and statistical | Included junior and middle grade trainees | 5440 (2005 anaesthetists, 978 junior/middle grade)  No denominator. | Psychological distress  Trauma response | GHQ-12  IES-R | Psychological distress (GHQ-12 score >3) = 44.2% across all cohorts | - | Covid-19, concerns about infection risk | - | - |
| McCrossan, R. et al. A qualitative study on attitudes to rest breaks during and after an overnight shift. 2017. | Anaesthesia: Conference abstract | Cross sectional observational  National | Not described | Mixed methods survey  Piloted in Wales then extended nationally | Quantitative and qualitative | Thematic analysis by two independent researchers | All trainee levels | 2172/3772 (57.5%) | Performance  Safety  Wellbeing | Self-reported | - | Fatigue | Negative attitudes towards rest on night shifts  Feeling undervalued. | - | Good rest facilities |
| Carey, C., Gale, T. C. E. and Evans, C. R. Recruitment to anaesthesia training posts during the COVID-19 pandemic and beyond. 2022. | Anaesthesia | Editorial | NA | NA | Opinion | - | Trainees – focus on ST3’s. | - | Stress | - | - | - | Anaesthetic recruitment issues: structure of interviews, lack of progression, bottleneck. ARCP’s, examinations | - | Feeling valued |
| Subramaniam, J. et al. Recruitment to higher specialty training in anaesthesia in the UK during the COVID-19 pandemic: a national survey. 2022. | Anaesthesia | Cross sectional observational  National | 1 month 10 days | Mixed methods survey: created by modified delphi method with pilot survey | Quantitative and qualitative | Exploratory and sentiment analysis | Applicants to ST3 | 536/1056 | Poor wellbeing Burnout | Self-reported | Poor work life balance  Leaving the specialty/medicine  Ill health | Lack of autonomy | Rotations, lack of registrar training jobs (75% not confident they will get a registrar post), tick box environment, unfair application process, overworked, covid, curriculum changes, organising own top up year, not appreciated/valued | - | Mindfulness initiatives (although not as effective as targeting issues) |
| Huniak, M. and Howells, E. Reducing stress and burnout among anaesthesia trainees with the use of regular peer-led meetings. 2018. | Anaesthesia: Conference | Cross sectional observational  Local | Unknown | Survey  Creation not described | Quantitative | Descriptive | Trainees and consultants | 28 total (13 trainees).  No denominator. | Morale | Self-reported | - | - | - | - | Peer meetings , departmental engagement |
| Richardson, E. and Choudhury, M. Steps to improve the wellbeing of our anaesthetic trainees at Guy's and St Thomas' NHS Foundation Trust, today and in the future. 2020. | Anaesthesia: Conference | Cross sectional observational  Local | Unknown | Survey  Creation not described | Quantitative and qualitative | Descriptive | Trainees (specific levels not discussed) | 40/75 | Burnout | Self-reported | - | Fatigue | Lack of rest areas, lockers, departmental socialisation | Going into LTFT, changing careers | - |
| O'Donovan, D., Leslie, D. and Stacey, M. Stress management teaching in anaesthetic trainees. 2017. | Anaesthesia: COnference | Pre-post interventional study  Local  Pre and post stress management teaching, stressful simulation and debrief | 1 day | Non stressed and stressed observations recorded: HR, time to pass scope and collision total | Quantitative | Descriptive | Trainees (specific levels not discussed) | Not described | Stress | Physiological observations: HR, time to pass scope and number of collisions | - | - | Stressful clinical situations | - | - |
| Looseley, A. et al. Stress, burnout, depression and work satisfaction among UK anaesthetic trainees; a quantitative analysis of the Satisfaction and Wellbeing in Anaesthetic Training study. 2019. | Anaesthesia | Cross sectional observational  Multi-regional | 4 months | Survey  Created in consultation with psychologist, piloted locally | Quantitative | SPSS  Catagorisation as high/low risk of stress, burnout, depression, work satisfaction and compared to baseline categories using Fishers exact test. Binary logistic regression to explore significant factors. Used to create odds ratios. | Trainees from all levels and non- training juniors | 397/619 | Stress: Psychological  Burnout  Depression  Work satisfaction | PSS (Perceived stress scale)  aMBI-HSS (abbreviated Maslach Burnout Inventory)  HANDS (Harvard national depression screening day scale)  ERI (effort reward imbalance) | High perceived stress: 37%  High depression risk: 18%  High burnout risk: 25% | Perceived stress: Low exercise levels (p0.01), being female (p<0.05)  Burnout risk: M sex (p0.055), not being a parent (p0.012), >3 days sickness (p0.016)  Depression: Not being a parent (p0.001), low exercise (p0.032), >3 days sickness (p0.032)  Imbalance of effort with reward | Additional nonclinical work significantly associated with stress, burnout, depression and low satisfaction (all p<0.05). Revising >30min/week significantly associated with burnout and depression risk. | - | - |
| Wainwright, E. et al. Stress, burnout, depression and work satisfaction among UK anaesthetic trainees: a qualitative analysis of in-depth participant interviews in the Satisfaction and Wellbeing in Anaesthetic Training study. 2019. | Anaesthesia | Qualitative phenomenological  Multi-regional | Not described | Semi structured interviews performed until data saturation by trained psychologist.  Purposeful sampling of participants identified from part 1 quantitative study.  Interview schedule created from literature review and piloted. | Qualitative | Thematic analysis: inductive. | Trainees (CT2-ST7) | 12/14  (10: finding things harder, 2: finding things easier) | Stress: Psychological  Burnout  Depression  Work satisfaction | Self-reported | - | Feeling unsafe | High nonclinical work load, ‘tick box’ environment, amount of on call/night shift work, cultural shift in societal view of doctors, rota gaps, difficulties with study leave, frequent rotations, pay cuts, anonymous decontextualised feedback | Support at home and at work, taking responsibility for self-care | Patient contact,  enabling good patient outcomes, learning technical skills, peer support, one to one training |
| Walsh, A. and Ghori, K. Stressors in anaesthesia - The experience of specialist registrars. 2011. | Irish Medical Journal | Cross sectional Observational  National | Unknown | Mixed methods survey  Creation not described | Quantitative and qualitative | Descriptive | Specialty registrars (ST1-5) | 62/100 | Stress | Self-reported | - | Fatigue | Inadequate time for research/administrative tasks, lack of ICU beds, working too many hours, inadequate staffing, inadequate time for breaks | - | Support from consultants |
| James, T. et al. A study of fatigue amongst staff, associate specialist and specialty doctors, Trust doctors and clinical fellows in anaesthesia in the UK and non-consultant hospital doctors in Ireland. 2019. | Anaesthesia: Conference | Cross sectional observational  National | 1 months | Survey  Creation not described | Quantitative | Descriptive | Clinical fellows, trust doctors, SAS | 352/928 | Wellbeing: Emotional | Self-reported | - | Fatigue | Not having rest facilities during shift and post shift for nights | - | - |
| Chan, V. and Misra, U. Subjective wellbeing survey of anaesthetic staff at a district general hospital. 2020. | Anaesthesia: Conference | Cross sectional observational  Local | Unknown | Mixed methods survey  Creation not described | Quantitative and qualitative | Descriptive | Trainees (+ consultants, SAS, ODP and nurses) | 94 total (15 trainees)  81% response rate. | Wellbeing | WHO-5 wellbeing index | ST3-7 WHO score 60.  CT1-3 WHO score 70.  SAS WHO scores 52 (lowest) | - | ARCPs, organisational culture, administrative tasks, long hours | - | - |
| Yentis, S. M. et al. Suicide amongst anaesthetists - an Association of Anaesthetists survey. 2019. | Anaesthesia | Cross sectional observational  National | 2 months | Mixed methods survey  Creation not described | Quantitative and qualitative | Descriptive | Anaesthetists from all grades | 3638 | Suicide | Reported | Suicide by grade: 53% consultant/ equivalent, 40% trainee /fellow, 6% specialist /trust/staff grade, 1% other  By sex: 5.6:1 M:F  Mode of death: majority anaesthetic medication (induction agent) | Relationship/ family issues, non-work-related police investigation, fatigue, pre-existing mental health or physical health issues, poor colleague/trust support | Examination failure | - | Debriefs  Peer support |
| Connal, S. Supporting trainee wellbeing during COVID-19: An essentials honesty box at Barnet General Hospital. 2021. | Anaesthesia: conference | Cross sectional observational  Local | Trail for 2 months followed by survey | Mixed methods survey  Creation not described | Quantitative and qualitative | Descriptive | Trainees | 20/35 | Wellbeing | Self-reported | - | - | - | - | Sense of community and comradery |
| Serou, N. et al. Surgical incidents and their impact on operating theatre staff: Qualitative study. 2021. | BJS Open | Qualitative phenomenological  Regional | Unknown | Semi structured interviews created from literature review and consultation with patient safety and qualitative research experts. Piloted for face validity. Performed by 1 researcher. | Qualitative | Reflexive inductive thematic analysis.  Coded by 5 researchers. | Anaesthetists, surgeons, operating theatre AHPs | 45 total (3 trainees)  No denominator | Wellbeing Stress | Self-reported | - | - | Surgical incidents, workplace investigations | - | - |
| McClelland et al. A national survey of the effects of fatigue on trainees in anaesthesia in the UK. 2017. | Anaesthesia | Cross sectional observational  National | 9 months | Mixed methods survey  Piloted in Wales then extended nationally | Quantitative and qualitative free text. | Descriptive | Trainees (specific levels not discussed) | 2,170/3,772  57.5% | Physical health  Psychological health | Self-reported | Personal relationship  Ability to do job Ability to manage exam revision/audits | Fatigue | Lack of on shift rest facilities and rest facilities for between shifts, opportunities for rest on night shifts | - | - |
| Henderson, M., Agaram, R. and Ryan, F. A survey of the stresses associated with training in obstetric anaesthesia. 2015. | Anaesthesia: Conference | Cross sectional observational  Local | Unknown | Mixed methods survey  Creation not described | Quantitative and qualitative free text | Descriptive | Trainees (specific levels not discussed) | 25  No denominator | Stress | Self-reported | - | Inexperience | Obstetric night shifts, clinical scenarios (GA for urgent C sections, pre-eclampsia/eclampsia, failed regional), poor communication, obstetric responsibility (high risk patients, ‘two-patients’), workload intensity, distant supervision | - | - |
| Frankling, C., Werpachowska, E. and Osborn, N. Survey of trainee anaesthetists' experience of general anaesthesia in obstetrics. 2018. | Anaesthesia: Conference | Cross sectional observational  Local | Unknown | Survey  Creation not described | Quantitative | Descriptive | Trainees ‘from all grades’ | 39/74 | Stress | Self-reported | - | - | Clinical scenarios (massive obstetric haemorrhage, GA for obstetric patients), high workload, inadequate training, lack of senior help, distractions in theatre suite | - | - |
| MacDonald, J. and Charles, R. A survey of trainee fatigue in Health Education Yorkshire and Humber. 2019. | Anaesthesia: Conference | Cross sectional observational  Regional | Unknown | Survey  Creation not described | Quantitative | Descriptive | Trainees (obstetric and anaesthetic) | 211/522 | Physical health  Mental health | Self-reported | Personal relationships  Ability to perform job and manage projects.  Patient safety (reduced ability to concentrate, handover and drug errors) | Fatigue | Lack of rest facilities | - | - |
| Deeley, A. S., Dryden, C. and Gwinnutt, J. Survey of trainees on pastoral care in anaesthetic training. 2018. | Paediatric Anaesthesia: Conference | Cross sectional observational  Regional | Unknown | Survey  Creation not described | Quantitative | Descriptive | Registrars | 36/50 | ‘Adverse effects’ | Self-reported | - | - | Critical incidents | - | - |
| Powell, L. et al. Tackling fatigue: A local approach. 2017. | Anaesthesia: conference | Case report  Local | Unknown | N/A | Opinion | N/A | Trainees (specific levels not discussed) | - | Fatigue | N/A | - | - | - | - | Trainee listening events |
| Mohabir, A. et al. Theatre staff morale at royal Berkshire NHS foundation trust. 2017. | Anaesthesia: Conference | Cross sectional observational  Local | Unknown | Survey  Creation not described | Quantitative | Descriptive | Trainees (in addition to consultants, and theatre staff) | Total 116 (18 trainees)  No denominator. | Morale | Self-reported | 44% reported poor self-morale and 39% reported poor departmental morale, 6% felt appreciated by Trust, 67% felt appreciated by department.  (Compared to 4% 20%, 28% and 100% respectively for consultants) | Not feeling appreciated | - | - | - |
| McCrossan, R., Stacey, K. and Redfern, N. Time to extinguish burnout. 2019. | Anaesthesia | Editorial | N/A | N/A | Opinion | N/A | All levels | - | Burnout | N/A | - | - | Irregular hours, nonclinical workload, changing rotas | Sense of job control | Mentoring, sense of teamwork |
| Tucker, P. and Byrne, A. The tiring anaesthetist. 2014. | Anaesthesia | Editorial | N/A | N/A | Opinion | N/A | All levels | - | Fatigue | N/A | - | Fatigue | On call shift burden | - | Fewer night shifts, shorter shifts, adequate rest times between shifts |
| Warren, J. et al. Trainee doctors' experiences of learning and well-being while working in intensive care during the COVID-19 pandemic: a qualitative study using appreciative inquiry. 2021. | BMJ open | Qualitative phenomenological  Local | 1 month | Semi structured interviews based on appreciative inquiry. Performed by peers. | Qualitative | Thematic analysis by 2 researchers | Trainees (foundation to ST8) | 40 total (32 anaesthetic trainees) | Wellbeing | Self-reported | - | Lack of autonomy | Covid infection and PPE concerns, intensity of rotas, emotional burden of work | Social support, self-care, sense of fulfilment, Maslow’s hierarchy of needs | Proactive leadership and induction, food, free parking, and rest facilities during covid |
| Cudlipp, J. Traumatic incidents in theatres and support for staff. 2021. | Anaesthesia: conference | Cross sectional observational  Local | Unknown | Survey  Creation not described | Quantitative | Descriptive | Theatre staff including anaesthetists | 137 total (24 anaesthetists) | Wellbeing | Self-reported | - | - | Surgical incidents | - | - |
| Kwanten, L. E. The UK anaesthesia workforce is in deepening crisis. 2021. | British journal of anaesthesia | Editorial | N/A | N/A | Opinion | N/A | All levels | - | - | - | - | - | Clinical pressures (ageing population), increased working hours/evening work, covid-19, decreased pay, backlog of elective cases, impact of junior doctor dispute | - | - |
| Tuthill, J. I. et al. Work-related stress amongst doctors in intensive care, anaesthetics, accident and emergency and general medicine. 2013. | Critical Care: conference | Cross sectional observational  Local | 6 weeks | Survey | Quantitative | Descriptive | ICU, anaesthetics, A+E and general medical trainees | 72 total (27 anaesthetists) | Stress: Work related | Health & Safety Executive Management Standards Indicator Tool | Stress level (1 highest amount, 5 lowest amount) anaesthetics:  3.4  Intensive care: 3.63 | - | Work demands | Peer support, staff relationships | - |
| RCoA. A report on the welfare, morale, and experiences of anaesthetists in training. The need to listen. 2017. | N/A | Cross sectional observational National | 1 year | Mixed methods survey + ‘listening events’ (focus groups)  Creation not described | Quantitative and qualitative free text and focus groups | Descriptive | Trainees of all grades | 2,312 (58% of all anaesthetists in training in UK)  200 trainees at listening events | Welfare  Morale  Burnout | Self-reported  Oldenburg burnout inventory | 78% experienced detrimental health due to employment  61% felt mental health negatively affected.  85% at higher risk of burnout  19% excessive levels of stress | Poor work life balance (n=211), , limited control/autonomy particularly with location | 75% have gone through a shift without sufficient hydration, 62% without a meal, filling rota gaps (average asked 6x per month) (n=66), late rota provision, system pressures (beds) (n=179), workload intensity (supporting other specialties e.g. cannulation), service provision vs training, examinations more difficult and not relevant (n=94), cost of training (n=70), assessment burden (n=209) (tick-box approach), rotations, long commutes, nonclinical workload, inadequate rest facilities, restricted food and drink outlets out of hours, no lockers, impact of junior doctor dispute (n=74), workplace culture (n=315), politics (n=123)  only 4% stated they felt valued by chief executive of hospital | LTFT for work-life balance, supportive senior trainees | Anaesthetic departments considered ‘welcoming and friendly’. Safe autonomous practice with appropriate supervision |
| RCoA. RCOA views from the frontline of anaesthesia during covid 19 April 2020 survey results. 2020. | NA | Cross sectional observational  National | 1 day | Mixed method survey  Creation not described | Quantitative and qualitative | Descriptive | All RCoA members including trainees | 2,174, unclear number of trainees | Mentally unwell  Physically unwell | Self-reported | - | - | Health concerns from lack of PPE, increased shift number and length | - | High team morale |
| RCoA. RCOA views from the frontline of anaesthesia during covid 19 May 2020 survey results. 2020. | NA | Cross sectional observational  National | 1 day | Mixed method survey  Creation not described | Quantitative and qualitative | Descriptive | All RCoA members including trainees | Not described | Mental distress  Morale | Self-reported | - | - | Health concerns from lack of PPE, difficulty taking leave, longer hours, lack of drug stocks (working with unfamiliar medication) | - | - |
| RCoA. RCOA views from the frontline of anaesthesia during covid 19 July 2020 survey results. 2020. | NA | Cross sectional observational  National | 6 days | Mixed method survey  Creation not described | Quantitative and qualitative | Descriptive | All RCoA members including trainees | 334, unclear number of trainees | Mental distress  Morale | Self-reported | - | - | Covid pressures and impact on progression and training, PPE and infection control concerns, difficulties taking annual leave, rota changes without agreement, lack of renumeration for extra work | - | - |
| RCoA. Respected, valued, retained. 2021. | NA | Cross sectional observational.  National. | 1 month | Mixed methods survey  Creation not described | Quantitative and qualitative | Descriptive | RCoA members including trainees. | 815 total (310 anaesthetists in training)  20% initial survey response rate. | Morale  Wellbeing  Fatigue | Self-reported | Leaving training:1/4 plan to lean NHS within 5 years, only 54% planning on working in NHS for entire career | Lack of work life balance, isolation from friends/family | Covid (3/10 said less likely to stay in NHS anaesthesia post covid) due to feeling under-appreciated, under-supported, overworked, trauma at work, poor training, curriculum changes. Lack of rota flexibility | making a difference.  Being able to work LTFT. | Less on call work, positive culture, portfolio careers would encourage retension  6% more likely to stay post covid due to supportive team, recognition from peers |
| AoA. Fatigue and anaesthetists. 2014. | NA | Expert consensus | N/A | N/A | - | - | - | - | Wellbeing  Fatigue | - | - | - | Out of hours work, shift patterns | Good sleep hygiene | Provision of rest breaks within and at the end of periods of work, availability of refreshments |
| Misra, U.  et al. Substance use disorder in the anaesthetist. 2022. | NA | Expert consensus | N/A | N/A | - | - | - | - | Substance misuse | - | - | Access to prescription and controlled drugs, poor levels of support, access to means (iv opioids, induction agents) | Excessive workload, stressful working environments with changing working patterns, poorly functioning teams, bullying and harassment | - | - |
| Shinde, S., et al. Guidelines on suicide amongst anaesthetists. 2019. | NA | Expert consensus | N/A | N/A | - | - | - | - | Suicide | - | - | A perfectionist personality type, male sex, ageing, fatigue, social or professional isolation, access to potentially lethal medications | Stress arising from complaints or bullying, high physical/mental demand, uncontrollable long hours/ shift working, compounded by examinations and frequent rotations for trainees, career progression | Support from friends/family | Coffee and a gas initiative |
